# Functional Electrical Stimulation (FES) in Adults with Neurological Disorders and Foot Drop: Orthotic and Therapeutic Effects in Short- and Long-Term Users

**DOI:** 10.3390/bioengineering13010071

**Published:** 2026-01-08

**Authors:** Niklas Bleichner, Merkur Alimusaj, Daniel W. W. Heitzmann, Andreas Stähle, Claudia Weichold, Cornelia Putz, Herta Flor, Frauke Nees, Sebastian I. Wolf

**Affiliations:** 1 Clinic for Orthopedics, Heidelberg University Hospital, 69118 Heidelberg, Germanymerkur.alimusaj@med.uni-heidelberg.de (M.A.); daniel.heitzmann@med.uni-heidelberg.de (D.W.W.H.); andreas.staehle@med.uni-heidelberg.de (A.S.); claudia.weichold@med.uni-heidelberg.de (C.W.); cornelia.putz@med.uni-heidelberg.de (C.P.); 2 Research Group Learning and Brain Plasticity in Mental Disorders, Department of Neuropsychology and Psychological Resilience Research, Central Institute of Mental Health, Medical Faculty Heidelberg University, 68159 Mannheim, Germany; herta.flor@zi-mannheim.de; 3 Institute of Medical Psychology, Ludwig-Maximilians-Universität (LMU Munich), 80336 Munich, Germany; frauke.nees@med.uni-muenchen.de

**Keywords:** neurological disorders, foot drop, functional electrical stimulation, gait, kinematics, spatio-temporal parameters, rehabilitation

## Abstract

Functional electrical stimulation (FES) is widely used to improve gait in individuals with neurological impairments; however, early responses in adults with congenital conditions, such as cerebral palsy, who are newly exposed to FES, remain poorly understood. This study investigated the orthotic and therapeutic effects of FES in short- and long-term users using standardized three-dimensional gait analysis. In this longitudinal study, short-term users (G1; n = 13; mean age 31.7 ± 18.1 years) were evaluated both without and with FES and followed over a 4–12-week insurance-covered trial period. Long-term users (G2; n = 11; mean age 32.2 ± 11.0 years), who had used FES for at least one year, were reassessed over a standardized 12-week interval. Linear mixed-effects models assessed the effects of FES and time, with subjects included as random effects to account for inter-individual variability. G1 showed significant therapeutic adaptations, including increased walking speed and step length and reduced step width, accompanied by decreased dorsiflexion during stance and swing, while no significant orthotic effects were observed. G2 demonstrated clear orthotic responses, such as increased dorsiflexion at heel strike and during swing and improved walking speed and step length, with minimal evidence of additional therapeutic adaptation. The initial reduction in dorsiflexion in G1 warrants further investigation. These findings suggest that evaluation timelines may need to be extended and that outcome measures beyond foot clearance should be considered, particularly given the heterogeneity and severity of congenital neurological conditions.

## 1. Introduction

Functional Electrical Stimulation (FES) is a commonly used treatment method for improving the gait of adults with neurological disorders characterized by upper motor neuron lesions and foot drop. It primarily addresses motor function related to gait [1] but also targets spasticity [2] as well as muscle strength and volume [3]. If the lower motor neurons and peripheral nerves remain intact, impaired muscles can be activated to restore motor function [4] and structural adaptations not only in the stimulated muscle but also in related musculature [5].

When applied over an extended period, the repetitive activation of the patient’s own muscles can promote neuroplasticity [6]. This neuroplasticity is considered a key mechanism underlying therapeutic benefits [7], with individuals demonstrating higher levels of plasticity achieving greater therapeutic improvements [8].

In the literature, the effects of FES treatment are divided into “orthotic” and “therapeutic” effects [1,6,9,10,11]. Orthotic effects denote the immediate effects of FES at the given time, i.e., gait performance without/with stimulation, whereas therapeutic effects stand for long-term improvements resulting from sustained FES use that persist in the absence of stimulation. These should be distinguished from short-term carry-over effects, which represent a temporary continuation of orthotic benefits after stimulation has stopped, but do not necessarily indicate lasting therapeutic change. The effects of FES and the time required for patients to experience its benefits vary substantially depending on the underlying neurological condition.

For adults with acquired neurological disorders, FES often yields promising results. Since their neuromuscular systems were previously intact, structural limitations are often less prominent and gait patterns may only show isolated impairments, allowing for more immediate orthotic effects and earlier functional improvements. In stroke patients, it has been shown that FES induces orthotic benefits, such as improved walking speed [12], with additional therapeutic benefits over time [9,13,14]. However, the evidence for enhanced gait kinematics remains weak [15]. Similarly, in spinal cord injury, positive orthotic [16,17] and therapeutic effects [18] have been reported.

In contrast to adults with acquired neurological disorders, individuals with congenital neurological disorders, including cerebral palsy (CP) or hereditary spastic paraplegia (HSP), may require longer treatment durations to achieve functional improvements. In patients with CP, the immediate orthotic effects of FES on walking speed and ankle dorsiflexion during swing are inconsistent, with some studies reporting no improvements in children [19] and adults [20]. However, after three months, therapeutic effects on walking speed have been observed in adults with CP [20], as well as after multiple years [21], indicating that improvements are possible but may require extended treatment durations. The fully developed neuromuscular system in adults with congenital-onset disorders, shaped by long-standing structural and functional adaptations, may thus necessitate longer intervention periods before meaningful changes emerge. Importantly, evidence directly comparing treatment responsiveness between adults with congenital-onset disorders and those with acquired neurological injuries remains scarce.

In children with CP, other studies indicate positive orthotic effects on ankle dorsiflexion during swing [2,22,23,24,25], although these effects are often accompanied by either a decrease or no change in walking speed [11,22,24]. While some studies report positive orthotic effects related to foot lift, early therapeutic outcomes in adults with CP can show a global shift toward a more plantarflexed foot position, yet at the same time faster walking speeds and positive subjective patient feedback [20].

In patients with multiple sclerosis (MS), FES can show positive orthotic effects on walking speed [9,26,27] and therapeutic effects within the first three months [28], with an offset afterwards, which may be attributed to the progressive nature of the disease [9]. On foot lift, similarly to adults with CP, over time, the foot position can show a small global shift towards a more plantarflexed foot position after eight weeks of treatment [28].

The observation times used to evaluate therapeutic effects are highly heterogeneous, typically ranging from three months to more than one year [8,9,14,20,27,29]. Some studies have observation times less than 3 months [2,22], whereas others only provide a single time point without clear information on the duration of treatment [24]. This variability makes it difficult to compare outcomes across studies and neurological conditions and complicates clinical decision-making, as short or inconsistent observation periods may fail to capture the full therapeutic potential of FES.

In clinical practice, FES systems are often prescribed with an initial trial period focused mainly on improvements in foot lift to determine eligibility for long-term reimbursement. However, this short trial period may fail to capture the full therapeutic potential of FES, especially for adults with congenital neurological disorders. Given the variability in response times across different neurological disorders, it is important to assess whether the standard evaluation period is sufficient and whether the exclusive emphasis on foot lift as a primary outcome measure should be reconsidered.

Despite existing studies, early responses to FES in adults with congenital neurological conditions remain poorly understood. Evidence on immediate orthotic effects and the timeline of therapeutic improvements is limited and inconsistent, leaving uncertainty about optimal evaluation periods and outcome measures. Consequently, this study aims to address existing gaps by analyzing a patient group during their 4- to 12-week trial period in clinical practice in comparison to long-term users. We intend to evaluate the longitudinal effects of TIME (T1 vs. T2, corresponding to the therapeutic effect) and FES CONDITION (without vs. with FES, corresponding to the orthotic effect) on short-term users (G1) during their trial period of 4 to 12 weeks, as well as long-term users (G2) over 12 weeks. We hypothesize that TIME will primarily alter gait parameters in short-term users, reflecting the therapeutic effect, whereas in long-term users, TIME will have little impact, with greater changes driven by FES, reflecting the orthotic effect.

## 2. Materials and Methods

### 2.1. Sample

Subjects included in the study were either retrospectively identified from our local pilot study database (S-112/2017) [20] or prospectively recruited in the Orthotics and Prosthetics Department of the Heidelberg University Orthopaedic Hospital. To maintain consistency and reliability, the prospective study was aligned with the retrospective pilot study in terms of inclusion criteria, outcome measures, and assessment protocols. All included patients provided written informed consent prior to inclusion. The study received approval from the Ethics Committee of the Medical Faculty of Heidelberg University (S-503/2022) and all methods were carried out in accordance with relevant guidelines and regulations (Declaration of Helsinki). The study was also registered with the German Clinical Trials Register (DRKS) under the registration number DRKS00034437.

The fitted FES systems included the Evomove (Evomotion GmbH, Lueneburg, Germany) or the L300Go and MyGait system (Otto Bock HealthCare Deutschland GmbH, Duderstadt, Germany) as their primary treatment device. All three devices consist of a stimulation unit that is attached to the shank and transcutaneously stimulates the Common Peroneal Nerve below the fibula head to activate the M. tibialis anterior and M. peroneus longus. The Evomove and the L300Go systems use accelerometer and gyroscope data to detect gait phases, while the MyGait system uses an instrumented sock, which transfers gait event signals (foot contact, foot-off) wirelessly to the main unit. For each participant, electrode placement, stimulation timing, and stimulation parameters were individually fitted and optimized by a certified orthotist according to the patient’s anatomy, gait pattern, and tolerance. Stimulation parameters were therefore adjusted on an individual basis and were not standardized across participants. To ensure optimal stimulation outcomes, each subject’s specific needs have been adjusted by a certified local orthotist with a four-week check-up for the newly treated patients and follow-up check-ups every six months for long-term users.

Inclusion criteria were: the ability to provide informed consent, age between 16 and 70 years, and the presence of a neurological disorder with upper motor neuron involvement causing foot drop. Detailed diagnostic and demographic characteristics of the study population are presented in the Section 3 (Section 3.1, Table 1). Participants were required to be able to walk independently for at least 15 min without the use of additional walking aids such as a cane or crutches.

Exclusion criteria included relevant comorbidities that could affect gait or FES response, specifically the presence of a structural pes equinus foot deformity with a plantarflexion contracture exceeding 5° and any previous surgical interventions or botulinum toxin injections in the leg muscles during the past six months.

### 2.2. Study Design

A within-subject repeated measures design was used for both groups. G1 included subjects starting FES treatment, and G2 included long-term users who had been using FES for at least one year. For both groups, conventional 3D gait analysis and clinical examination were conducted at study entry (T1) and at a follow-up assessment (T2). For G1, the duration of the test phase varied between 4 and 12 weeks depending on the reimbursed time provided by the individual’s insurance, while for G2, T2 was conducted at a fixed interval of 12 weeks. Three-dimensional gait analysis was performed on level ground and walking up and down a 10° inclined ramp in fixed order, first without FES, followed by with FES. In addition, during each visit, subjects completed questionnaires assessing quality of life, dual-task challenges in daily life, and the frequency of toe dragging and falls. However, these questionnaire data were not available for the retrospectively included participants in G1.

For conventional 3D gait analysis, a Vicon System (Oxford, UK) consisting of twelve cameras (T40s, @120Hz) was used. Reflective, skin-mounted markers were placed on each subject following the Plug-In-Gait (PiG) protocol [30,31,32]. Ground reaction forces were measured using two force plates (1080Hz, AMTI, Inc., Watertown, MA, USA) discreetly embedded in the walkway. Gait parameters were calculated as the average of at least seven valid gait cycles per participant. For kinetic analyses, only gait cycles with valid force plate contact were included. Joint kinematics and kinetics were calculated using the conventional inverse dynamics approach with PiG software (Vicon, Oxford, UK), normalized to body weight, and time-normalized to the gait cycle (0–100%). Positive ankle work was calculated by integrating the positive portion of the ankle power over time across the gait cycle, representing push-off energy in J/kg. Outcome measures

Ankle kinematics and kinetics were calculated to quantify foot lift and push-off energy, and for overall adjustments in gait kinematics, the gait profile score (GPS) was calculated [33]. Additionally, spatio-temporal parameters such as walking speed, step length, and step width were evaluated to assess overall gait function.

Questionnaires included the 12-Item Short Form Health Survey (SF-12) [34] focusing on the Physical Component Summary (PCS) and Mental Component Summary (MCS) for quality of life; the Dual-task Impact on Daily-living Activities Questionnaire (DIDA-Q) [35] to assess patients dual-task difficulties during everyday-life activities; and a 5-point ordinal scale to evaluate toe dragging and falling, with higher scores indicating more frequent incidents [2].

### 2.3. Statistical Analyses

Linear mixed models (LMMs) were conducted for each group to evaluate the effects of FES CONDITION (orthotic effect: without vs. with FES), TIME (therapeutic effect: T1 vs. T2), and their interaction on gait parameters. Both FES CONDITION and TIME were treated as fixed effects, while subjects were included as random effects to account for inter-individual variability. Post hoc comparisons of estimated marginal means were conducted using Bonferroni-corrected pairwise t-tests to adjust for multiple comparisons with mean differences, *p*-values, and 95% confidence intervals (CIs). Due to personal circumstances unrelated to the treatment device, some participants did not complete all sessions, resulting in unequal group sizes across time points. Furthermore, differences between the groups reflect the nature of the difficulties in clinical practice. Summarizing, this led to the following group sizes: G1@T1 = 13; G1@T2 = 11; G2@T1 = 11; G2@T2 = 9. LMMs were chosen as they accommodate unbalanced designs and missing data without listwise deletion. Model assumptions were evaluated by inspecting residual Q-Q plots and performing Shapiro–Wilk tests for normality. While one model showed a significant Shapiro–Wilk result (*p* = 0.026), visual inspection and non-significant results in the remaining models indicated no substantial violation of the normality assumption. To compare the questionnaire outcomes of group G2 between T1 and T2, the Wilcoxon signed-rank test has been performed. For all parameters, the significance level was set at *p* < 0.05, and statistical analyses were performed using SPSS (version 27; SPSS, Chicago, IL, USA). Baseline demographic and clinical characteristics were summarized descriptively and are presented in the Section 3 (Section 3.1, Table 1).

## 3. Results

### 3.1. Demographics

In total, for both groups, 24 subjects with neurological impairments at the upper motor neuron level, such as cerebral palsy, incomplete spinal cord injury, multiple sclerosis, hereditary spastic paraplegia, stroke, and traumatic brain injury, who exhibited foot drop, were included in this analysis. Table 1 presents their demographic data divided into the groups short-term (G1) and long-term users (G2).

### 3.2. Group 1 (Short-Term Use)

In Group 1, the linear mixed model revealed significant main effects for TIME but not for FES CONDITION (Table 2 and Table 3). Pairwise comparison showed a significant decrease over time in maximal dorsiflexion during stance of –1.31 ± 0.57° (*p* = 0.029; 95% CI: –2.469 to –0.146) and in swing of –2.49 ± 0.74° (*p* = 0.002; 95% CI: –3.997 to –0.974) (Table 4 and Table 5). In terms of spatio-temporal parameters, speed demonstrated a significant increase of 0.07 ± 0.02 m/s (*p* = 0.006; 95% CI: 0.022 to 0.117) over time similar to step length 0.02 ± 0.01 m (*p* = 0.034; 95% CI: 0.001 to 0.035) while step width showed a decrease of –1.04 ± 0.37 cm (*p* = 0.009; 95% CI: –1.794 to –0.279). No significant main effects were found for FES CONDITION or the interaction of TIME and FES CONDITION in Group 1.

### 3.3. Group 2 (Long-Term Use)

In Group 2, the linear mixed model revealed significant main effects for TIME and FES CONDITION (Table 2 and Table 3). Pairwise comparison showed time-related increases in maximal dorsiflexion during stance of 2.37 ± 0.60° (*p* = 0.001; 95% CI: 1.133 to 3.603) and in push-off energy of 0.02 ± 0.01 J/kg (*p* = 0.009; 95% CI: 0.005 to 0.034) (Table 4). In terms of kinematics, FES led to a significant increase in dorsiflexion at heel strike of 2.80 ± 1.03° (*p* = 0.012; 95% CI: 0.677 to 4.915) and in maximal dorsiflexion during swing of 2.05 ± 0.91° (*p* = 0.034; 95% CI: 0.168 to 3.924) (Table 4). For spatio-temporal parameters, FES significantly increased walking speed of 0.06 ± 0.02 m/s (*p* = 0.004; 95% CI: 0.021 to 0.096) as well as step length of 0.03 ± 0.01 m (*p* = 0.002; 95% CI: 0.011 to 0.042) (Table 5). No significant main effects were found for the interaction of TIME and FES CONDITION in Group 2 as well.

Questionnaire results for Group 2 did not show significant changes over time (all *p* > 0.345). Median scores at T1 were: DIDA-Q: 39 (IQR: 29 to 45), SF-12 Physical Component Score (PCS): 51.5 (IQR: 44.9 to 56.6), SF-12 Mental Component Score (MCS): 40.6 (IQR: 36.1 to 41.5), toe dragging: 2 (IQR: 2 to 3.5), and falling: 2 (IQR: 1.5 to 2.5).

## 4. Discussion

This study examined TIME (therapeutic effect) and FES CONDITION (orthotic effect) in short-term (G1) and long-term (G2) FES users. In G1, therapeutic effects included a reduction in ankle dorsiflexion but improvements in walking speed, step length, and step width, with no significant orthotic effects. In contrast, G2 showed primarily orthotic improvements in dorsiflexion and spatio-temporal parameters, with minimal additional therapeutic gains.

### 4.1. Group 1 (Short-Term Users)

In G1, a significant TIME-related decline in maximum dorsiflexion during stance and swing resulted in a more plantarflexed foot position, with no significant orthotic effects (Table 2 and Table 3). This initial worsening aligns with our pilot study in adults with CP [20]. Although the literature generally reports neutral or positive effects on foot lift [1], raw ankle angle data in children with CP show similar early declines in dorsiflexion without FES [22,25], which later reverse after several months of FES use [25].

This early deterioration may reflect deeply ingrained compensatory gait patterns in congenital neuropathies such as CP. One possible mechanism for the observed early decline in dorsiflexion is abnormal muscle activation in congenital upper motor neuron lesions. Individuals with cerebral palsy show increased agonist–antagonist co-activation during gait [36] and altered reflex activity with impaired reciprocal inhibition [37], which may contribute to the early reductions in dorsiflexion when initiating FES. Lifelong adaptations to structural and functional limitations and impaired neuromuscular control contribute to persistent abnormal gait [38]. The introduction of FES may initially disrupt these established strategies as the neuromuscular system adapts to a novel external stimulus

Despite reduced dorsiflexion, significant improvements in walking speed, step length, and step width suggest enhanced dynamic stability and overall gait efficiency. Importantly, although the reduction in maximum dorsiflexion reached statistical significance, its functional impact should be interpreted with caution. The observed decrease remained within a relatively small angular range and occurred alongside clinically meaningful improvements in global gait parameters. The reduction in step width may further indicate improved mediolateral control, consistent with reports on balance, dual-task performance, and gait symmetry [39,40,41]. These findings highlight that the early therapeutic benefits of FES may manifest primarily in global gait parameters, even when local ankle kinematics show transient declines.

### 4.2. Group 2 (Long-Term Users)

In contrast, G2 improvements were primarily orthotic (Table 2, Table 3, Table 4 and Table 5). FES increased dorsiflexion at heel strike and during swing, supporting controlled foot placement and adequate toe clearance, which are critical for reducing the risk of tripping [2,25,42].

Walking speed and step length also increased, which is in line with previous research on long-term FES use [1,6,9,13,43].

No outcomes of the self-report questionnaires changed significantly over the same period. The overall stability in both subjective and objective measures, except for the small TIME-related changes in maximum dorsiflexion during stance and push-off energy, suggests that these ongoing biomechanical changes are subtle and of limited functional relevance. The absence of corresponding changes in spatiotemporal gait parameters or self-reported outcomes indicates that these TIME-related effects do not translate into further meaningful functional improvements. This consistency supports the interpretation that, for long-term users in G2, functional gains are primarily attributable to the orthotic effects of FES rather than continued time-dependent therapeutic adaptation, indicating a potential plateau in long-term neuro-biomechanical change. It should be noted, however, that the self-report questionnaires may not be sensitive enough to detect further time-dependent changes.

### 4.3. Clinical Implication

Differences between groups emphasize the importance of treatment duration. Short-term users may show early therapeutic gains in gait parameters despite the deterioration in ankle kinematics, whereas long-term users demonstrate robust orthotic benefits.

Clinically, minimal differences in walking speed are defined as changes between 0.05 and 0.082 m/s [44,45], and clinically relevant increases in maximum dorsiflexion during swing range from 2.3° to 5.6° [22,24].

In G1, the therapeutic effect on walking speed (+0.07 m/s) indicates a clinically meaningful improvement over time. However, this is accompanied by a clinically relevant deterioration in maximum dorsiflexion during swing (−2.49°). In G2, the orthotic effects, including an improvement of 2.80° at heel strike, 2.05° in maximum dorsiflexion during swing, and a 0.06 m/s increase in walking speed, are at the threshold of clinical relevance.

The initial worsening of global foot position in G1, especially among patients with congenital neuropathies such as CP, has received little attention in the literature. However, it is highly relevant for understanding time-dependent therapeutic effects of FES and for informing reimbursement decisions. Given that typical clinical observation periods for FES in Germany are limited to 4–12 weeks, our results indicate that such short intervals may miss meaningful improvements in patients with complex congenital impairments. Extending treatment and observation periods to 6–12 months may be necessary to fully reveal therapeutic effects on dorsiflexion and ankle kinematics, even though improvements in walking speed can occur earlier, ranging from 4 weeks to 12 months [20,21,46]. Further follow-up studies are needed to clarify the timeline of dorsiflexion changes, as current evidence provides limited insight into the specific effects of FES on ankle function in adults with congenital conditions like CP.

Consequently, evaluation frameworks should consider both the neurological condition and impairment severity. FES treatment protocols should be individualized based on diagnosis (congenital vs. acquired) and patient characteristics (adult vs. child), ensuring that treatment duration matches the expected timeline for meaningful improvements in gait patterns and dynamic stability. Additionally, since other beneficial effects of FES, like dual-task performance with improved cognition rate [39] or obstacle avoidance [47], have been demonstrated, evaluations should consider a broader range of outcome measures beyond foot lift alone.

### 4.4. Limitations and Future Directions

Due to the small and inhomogeneous cohorts, the generalizability of the results is limited, and the potential lack of statistical power may have led to undetected effects. Additionally, the two groups are independent and have different baseline characteristics. G1 primarily consists of individuals with congenital neuropathies, which may have influenced the observed outcomes. Although Bonferroni corrections were applied for post hoc pairwise comparisons, no further adjustment for multiple outcome measures was performed, and therefore, a risk of Type I error across the multiple dependent variables remains. Additionally, variability in electrode placement and stimulation may have influenced individual responses, despite fitting by certified orthotists. Device type was selected jointly by the patient and a certified orthotist based on individual needs and preferences; therefore, it was not controlled experimentally and was not tested as a covariate, also due to the limited sample size. We did not monitor daily wear time or activity levels, both of which may influence therapeutic adaptation and overall FES efficacy.

Furthermore, cohort bias may be present. Although G1 participants are positive responders to FES and wish to continue treatment, they remain in the test phase, with weak effects on foot lift and possibly no further improvement over time. Therefore, longer-term follow-up measurements in both groups, along with a clearer stratification by neurological diagnosis, are necessary to better understand the interaction between TIME and FES across different populations.

Motivation bias may also have influenced the results, as study participation could have increased engagement and familiarity with the measurement procedures. Such habituation effects may partly explain the additional therapeutic improvements observed in G2.

## 5. Conclusions

FES can improve gait function and dynamic stability in adults with neurological impairments. Short-term users show early therapeutic improvements in global gait parameters despite an initial worsening in foot position. Long-term users demonstrate predominantly orthotic benefits, with improvements in dorsiflexion, walking speed, and step length. Notably, dorsiflexion improvements were observed only in users treated for at least 12 months, whereas no gains were seen in the 4- to 12-week period, highlighting that this intermediate time frame still requires further investigation. These findings underscore the critical importance of the initial test phase, particularly for adults with congenital neurological conditions, and suggest that evaluation periods for treatment reimbursement and relevant outcome measures beyond foot lift should be reconsidered. Extending evaluation periods to at least 6–12 months may be necessary to allow patients to fully realize the therapeutic potential of FES.

## Figures and Tables

**Table 1:** Demographics of included patients (SD: standard deviation) for G1 and G2.

Characteristic	Group 1 (Short-Term Users)	Group 2 (Long-Term Users)
Total	13	11
Diagnosis	9 CP (4 uni-, 5 bilateral), 1 incomplete SCI, 1 HSP, 1 Stroke, 1 TBI	6 CP (3 uni-, 3 bilateral), 4 incomplete SCI, 1 MS
Gender	7 f, 6 m	8 f, 3 m
Age [years]	31.7 ± 18.1	32.2 ± 11.0
Height [cm]	169.0 ± 10.1	168.1 ± 9.2
Weight [kg]	62.7 ± 15.7	68.4 ± 17.4
Foot deformity	5 equinus, 6 flatfoot, 2 no deformity	1 equinus, 5 flatfoot, 1 pes calcaneus, 4 no deformity

CP: cerebral palsy; SCI: spinal cord injury; MS: multiple sclerosis; HSP: hereditary spastic paraplegia; TBI: traumatic brain injury; f: female; m: male.

**Table 2:** Mean values (SD) of gait variables of short- (G1) and long-term users (G2) with linear mixed model results with TIME and FES as fixed effects and subjects as random effects (G1T1 = 13; G1T2 = 11; G2T1 = 11; G2T2 = 9).

			G1								*p*		
Kinematics and kinetics	TD		T1 w/o FES		T1 w/FES		T2 w/o FES		T2 w/FES		TIME	FES	TIME × FES
GPS [°]			9.11	(3.24)	8.87	(2.93)	9.65	(1.97)	9.25	(1.73)	0.367	0.454	0.849
Max. dorsiflexion stance [°]	19.26	(2.87)	16.34	(3.57)	16.50	(3.81)	14.63	(4.31)	14.97	(3.18)	**0.029**	0.650	0.876
Dorsiflexion at heel-strike [°]	6.68	(2.68)	−1.93	(5.69)	−0.72	(5.87)	−3.84	(6.11)	−2.48	(7.25)	0.057	0.140	0.926
Max. dorsiflexion swing [°]	8.17	(2.80)	0.65	(4.82)	1.99	(5.33)	−1.51	(5.11)	−0.61	(7.02)	**0.002**	0.125	0.760
Push-off energy [J/kg]	0.32	(0.07)	0.18	(0.05)	0.16	(0.06)	0.16	(0.06)	0.16	(0.07)	0.204	0.125	0.101
			G2								*p*		
Kinematics and kinetics	TD		T1 w/o FES		T1 w/FES		T2 w/o FES		T2 w/FES		TIME	FES	TIME × FES
GPS [°]			8.22	(2.29)	8.29	(2.58)	8.38	(1.68)	8.28	(1.91)	0.927	0.954	0.775
Max. dorsiflexion stance [°]	19.26	(2.87)	18.82	(7.86)	20.10	(8.99)	21.07	(8.31)	21.95	(7.53)	**0.001**	0.070	0.728
Dorsiflexion at heel-strike [°]	6.68	(2.68)	−1.57	(8.97)	2.34	(7.57)	−1.02	(8.48)	0.66	(7.47)	0.694	**0.012**	0.289
Max. dorsiflexion swing [°]	8.17	(2.80)	2.70	(8.94)	5.82	(8.31)	3.66	(8.34)	4.63	(6.66)	0.392	**0.034**	0.250
Push-off energy [J/kg]	0.32	(0.07)	0.20	(0.12)	0.19	(0.11)	0.19	(0.10)	0.17	(0.09)	**0.009**	0.062	0.388

TD: typically developed, FES: functional electrical stimulation, GPS: gait profile score; w/o: without, w/: with; bold values indicate statistically significant changes.

**Table 3:** Mean values (SD) of spatio-temporal parameters of short- (G1) and long-term users (G2) with linear mixed model results with TIME and FES as fixed effects and subjects as random effects (G1T1 = 13; G1T2 = 11; G2T1 = 11; G2T2 = 9).

			G1								*p*		
Spatio-temporal parameters	TD		T1 w/o FES		T1 w/FES		T2 w/o FES		T2 w/FES		TIME	FES	TIME × FES
Speed [m/s]	1.39	(0.17)	1.04	(0.23)	1.01	(0.22)	1.13	(0.22)	1.14	(0.19)	**0.006**	0.626	0.399
Step length [m]	0.75	(0.08)	0.60	(0.07)	0.61	(0.07)	0.64	(0.06)	0.65	(0.06)	**0.034**	0.353	0.913
Step width [cm]	9.09	(1.92)	9.24	(4.40)	9.25	(4.40)	8.30	(4.15)	7.75	(4.43)	**0.009**	0.455	0.446
			G2								*p*		
Spatio-temporal parameters	TD		T1 w/o FES		T1 w/FES		T2 w/o FES		T2 w/FES		TIME	FES	TIME × FES
Speed [m/s]	1.39	(0.17)	1.06	(0.19)	1.15	(0.17)	1.09	(0.15)	1.12	(0.17)	0.094	**0.004**	0.149
Step length [m]	0.75	(0.08)	0.61	(0.10)	0.65	(0.10)	0.61	(0.06)	0.63	(0.06)	0.293	**0.002**	0.150
Step width [cm]	9.09	(1.92)	9.00	(1.86)	8.02	(2.39)	7.86	(3.11)	7.97	(3.11)	0.114	0.269	0.170

TD: typically developed, FES: functional electrical stimulation; w/o: without, w/: with; bold values indicate statistically significant changes.

**Table 4:** Significant pairwise comparisons of gait variables of the linear mixed model results for short- (G1) and long-term users (G2).

	G1									
	TIME					FES				
Kinematics and kinetics	Mean diff.	SD	*p*-value	95 CI		Mean diff.	SD	*p*-value	95 CI	
Max. dorsiflexion stance [°]	−1.31	(0.57)	**0.029**	−2.469	−0.146	0.25	(0.55)	0.650	−0.871	1.376
Max. dorsiflexion swing [°]	−2.49	(0.74)	**0.002**	−3.997	−0.974	1.13	(0.72)	0.125	−0.332	2.586
	G2									
	TIME					FES				
Kinematics and kinetics	Mean diff.	SD	*p*-value	95 CI		Mean diff.	SD	*p*-value	95 CI	
Max. dorsiflexion stance [°]	2.37	(0.60)	**0.001**	1.133	3.603	1.08	(0.57)	0.070	−0.097	2.261
Dorsiflexion at heel-strike [°]	0.43	(1.08)	0.694	−1.783	2.641	2.80	(1.03)	**0.012**	0.677	4.915
Max. dorsiflexion swing [°]	0.83	(0.96)	0.392	−1.131	2.793	2.05	(0.91)	**0.034**	0.168	3.924
Push-off energy [J/kg]	0.02	(0.01)	**0.009**	0.005	0.034	−0.01	(0.01)	0.062	−0.027	0.001

FES: functional electrical stimulation, bold values indicate statistically significant changes.

**Table 5:** Significant pairwise comparisons of spatio-temporal parameters of the linear mixed model results for short- (G1) and long-term users (G2).

	G1									
	TIME					FES				
*Spatio-temporal parameters*	Mean diff.	SD	*p*-value	95 CI		Mean diff.	SD	*p*-value	95 CI	
Speed [m/s]	0.07	(0.02)	**0.006**	0.022	0.117	−0.01	(0.02)	0.626	−0.057	0.035
Step length [m]	0.02	(0.01)	**0.034**	0.001	0.035	0.01	(0.01)	0.353	−0.009	0.023
Step width [cm]	−1.04	(0.37)	**0.009**	−1.794	−0.279	−0.27	(0.36)	0.455	−1.000	0.458
	G2									
	TIME					FES				
*Spatio-temporal parameters*	Mean diff.	SD	*p*-value	95 CI		Mean diff.	SD	*p*-value	95 CI	
Speed [m/s]	0.03	(0.02)	0.094	−0.006	0.072	0.06	(0.02)	**0.004**	0.021	0.096
Step length [m]	0.01	(0.01)	0.293	−0.008	0.025	0.03	(0.01)	**0.002**	0.011	0.042

FES: functional electrical stimulation, bold values indicate statistically significant changes.

## Data Availability

The data supporting this study cannot be publicly shared due to ethical and privacy restrictions. However, the data will be made available from the corresponding author upon reasonable request.

## Abbreviations

The following abbreviations are used in this manuscript:

CIs	Confidence intervals
CP	Cerebral palsy
DIDA-Q	Dual-task Impact on Daily-living Activities Questionnaire
DRKS	German Clinical Trials Register
FES	Functional electrical stimulation
f	Female
G1	Short-term users
G2	Long-term users
GPS	Gait profile score
HSP	Hereditary spastic paraplegia
LMMs	Linear mixed models
MCS	Mental Component Summary
MS	Multiple sclerosis
m	Male
OE	Orthotic effect
PCS	Physical Component Summary
PiG	Plug-In-Gait
SF-12	The 12-Item Short Form Health Survey
SD	Standard deviation
TE	Therapeutic effect
TD	Typically developed
w/	With
w/o	Without
